# Effect of Brain-to-Skull Conductivity Ratio on EEG Source Localization Accuracy

**DOI:** 10.1155/2013/459346

**Published:** 2013-04-17

**Authors:** Gang Wang, Doutian Ren

**Affiliations:** ^1^Key Laboratory of Biomedical Information Engineering of Ministry of Education, Institute of Biomedical Engineering, School of Life Science and Technology, Xi'an Jiaotong University, 28 Xianning West Road, Xi'an, Shaanxi 710049, China; ^2^School of Public Management, Northwest University, 1 Xuefu Avenue, Xi'an, Shaanxi 710127, China

## Abstract

The goal of this study was to investigate the influence of the brain-to-skull conductivity ratio (BSCR) on EEG source localization accuracy. In this study, we evaluated four BSCRs: 15, 20, 25, and 80, which were mainly discussed according to the literature. The scalp EEG signals were generated by BSCR-related forward computation for each cortical dipole source. Then, for each scalp EEG measurement, the source reconstruction was performed to identify the estimated dipole sources by the actual BSCR and the misspecified BSCRs. The estimated dipole sources were compared with the simulated dipole sources to evaluate EEG source localization accuracy. In the case of considering noise-free EEG measurements, the mean localization errors were approximately equal to zero when using actual BSCR. The misspecified BSCRs resulted in substantial localization errors which ranged from 2 to 16 mm. When considering noise-contaminated EEG measurements, the mean localization errors ranged from 8 to 18 mm despite the BSCRs used in the inverse calculation. The present results suggest that the localization accuracy is sensitive to the BSCR in EEG source reconstruction, and the source activity can be accurately localized when the actual BSCR and the EEG scalp signals with high signal-to-noise ratio (SNR) are used.

## 1. Introduction

The electroencephalogram (EEG) measures scalp electrical potential which is propagated from neuronal source activity within the brain through the head volume conductor. The EEG signals can be recorded on the scalp of human head via appropriate electrodes, and they provide important information about brain electrical activity. These signals have been widely applied in neuroscience research [1–3] and used in clinical applications [4–6]. The EEG source localization techniques are the key points to solve these problems, and a number of efforts have been made to solve the so-called EEG inverse problem which aims at reconstructing brain electrical activity from scalp EEG measurements. In the majority of the EEG source localization methods, a piecewise homogenous head model is used to represent the physical properties of the human head volume conductor. This model usually consists of three compartments (brain, skull and scalp), which are segmented from MR images. Subsequently, the conductivities of different tissues are assigned to each compartment [7]. These conductivity values play a critical role in determining the relationship between the recorded scalp potentials and neuronal currents within the brain. In these head models, it is usually assumed that the scalp has the same conductivity as the brain, while the skull has a much lower conductivity. In addition, only the relative conductivities are considered when source location is concerned and the absolute strength of source is not. Thus, it is very important to specify the brain-to-skull conductivity ratio (BSCR) in EEG source localization.

Many attempts have been made to estimate the BSCR, and large varieties of this ratio have been reported. Rush and Driscoll [8] identified that the resistivity of skull was 80 times greater than the resistivity of brain and scalp, by employing an electrolytic tank to measure the impedance of the human skull. Cohen and Cuffin [9] indicated that the conductivity ratio should be 80 by using a combined analysis of the EEG and the magnetoencephalogram (MEG) recordings evoked by the same stimulus in a spherical model of the head. Subsequently, this conductivity ratio was widely accepted and used by researchers. However, in the past decade there have been many debates with regard to the value of BSCR. Oostendorp et al. [10] conducted in vivo experiments by using a boundary element head model and in vitro experiments to estimate the conductivity ratio, and both methods suggested a different BSCR value of 15. Recently, Lai et al. [11] employed a cortical imaging technique based on a spherical head model to estimate the human BSCR as 24.8 ± 6.6 from simultaneously recorded intra- and extracranial potentials in 5 epilepsy patients. Zhang et al. [7] further suggested the ratio to be 18.7 ± 2.1 by using accurate finite element modeling and simultaneous intra- and extracranial recordings in two epilepsy patients.

Studies also have been implemented in order to investigate the influence of conductivity ratio on EEG source localization accuracy. Awada et al. [12] used a 2-dimensional (2D) finite element model to examine the sensitivity of white matter, gray matter, cerebrospinal fluid, skull, and fat on EEG source localization and the effect of modeling errors on EEG source localization in noise-free condition. They found that the skull conductivity was the most sensitive factor in EEG source localization, and furthermore, the source location errors due to the skull conductivity were, on average, 5 mm with a maximum of 13 mm. Moreover, Huiskamp et al. [13] studied the localization errors from three dipole sources with different locations in a realistic geometry model when skull conductivity was either over- or underestimated. It was reported that the dipole location errors were around 10 mm in the context of considering noise-free measurements. Although these studies indicated that the misspecified BSCRs would result in large source localization errors, no systematic study has been reported to explore the relationships between BSCR and EEG source localization accuracy.

In this study, we focused on elaborating the effect of different BSCRs on EEG source localization accuracy by using huge amounts of dipole sources on the cortical surface and a 3-dimensional (3D) head model based on realistically shaped head volume conductor. We selected four BSCRs used previously: 15, 20, 25, and 80. Then, we evaluated the relationships between these BSCRs and EEG source localization accuracy by using an experimental protocol. For each dipole source, the scalp EEG was simulated by forward calculation when one of four BSCRs was assigned. The source reconstruction was performed by using the single equivalent current dipole (ECD) model for each different BSCR. The accuracy of EEG source localization was assessed by comparing the estimated sources to the simulated sources. Eventually, the effect of BSCR on EEG source localization accuracy was derived from the results of comparisons among these EEG source localizations by different BSCRs.

## 2. Materials and Methods

In order to evaluate the relationships between different BSCRs and EEG source localization accuracy, we employed four previously used BSCRs: 15, 20, 25, and 80. For the head modeling, a three-shell realistically shaped piece-wise homogeneous head volume conductor was employed, which contained three compartments (brain, skull, and scalp) [14]. The anatomical magnetic resonance (MR) images of a human subject (256 slices, a field of view of 256 mm, matrix size: 256 × 256, voxel size: 1 × 1 × 1 mm^3^) were acquired on a GE Signa machine. The surfaces (skin layer, outer skull layer, and inner skull layer) separating the three compartments were segmented from a set of high-resolution T1-weighted MR images of this subject using Curry software (V6, Compumedics, Charlotte, NC). The skin layer contained 1473 nodes and 2942 triangular elements. The outer skull layer consisted of 1324 nodes and 2644 triangular elements. The inner skull layer comprised 1872 nodes and 3740 triangular elements. The conductivities of the scalp and the brain were assumed to be 0.33 S/m. The conductivity of the skull was determined by the BSCRs mentioned above. Then, conductivity values were assigned to each of the compartments and a boundary element method (BEM) model was constructed from these segmentation results.

A 31-electrode setting was based on the modified 10/20 system configuration [15]. A single dipole source was used to represent the cortical neural activity. In this study, around 8000 equivalent current dipole sources were employed, and these dipoles were evenly placed over the folded cortical surface reconstructed from the MR images, and the orientation of each dipole was assumed to be perpendicular to the local cortical patch [16, 17]. 

The EEG forward calculation can present electrode measurements as a function of the source distribution. The forward model used in this study can be described with the equation as

(1) $$\begin{array}{c} f=L\cdot q, \end{array}$$

where *f* denotes the measurement vector of the simulated scalp EEG potentials and *q* represents the dipole column vector containing location and strength information of the dipole sources. *L* is the lead field matrix through which the measurement vector is linked with the dipole sources [18]. In the forward computation, both BSCRs and BEM model were involved in this lead field matrix. When calculating the simulated EEG signals, the average reference was used to determine all electrode potentials. Since the dipole source orientations were fixed, there was only one dipole strength which needed to be specified for each dipole location. For each of the dipoles on the folded cortical surface, the scalp potentials at 31 electrode locations were simulated using the BEM-based forward calculation [14] by assigning a certain conductivity ratio of four BSCRs. This assigned BSCR was considered as the correct ratio, and the other three BSCRs were assumed to be the incorrect ratios when the inverse calculation was performed. In the case of considering noise-free EEG measurements, these scalp potentials were referred to as the actual scalp EEG signals and were utilized as inputs to the following inverse calculations for investigating the influence of different BSCRs on EEG source localization accuracy.

In the EEG inverse modeling, the single ECD model [19] was used to estimate the location and strength of dipole source on the cortical surface from each scalp EEG measurement. The best-fitting dipole can be determined by minimizing the residual error between the simulated scalp EEG measurement and the forward-calculated measurement

(2) $$\begin{array}{c} \Delta^{2}={\left|M-Lq\right|}^{2}, \end{array}$$

where *M* denotes the simulated EEG measurement data, *q* is dipole component vector, and *L* is the lead field matrix which describes the transfer function from dipole components to the simulated scalp EEG measurements. The BEM model was also used in the inverse calculation. Solution space was defined on a surface source distribution which comprised around eight thousand current dipole sources evenly positioned on the folded cortical surface. Additionally, the dipole sources were constrained to predefined orientations which were perpendicular to the local cortical source surface. Thus, only one dipole strength should be estimated for each dipole source. The residual error was minimized using a Levenberg-Marquardt algorithm [20] to find the location and strength of a best-fitting dipole. In this study, only dipole location information was considered to be important since the location of neural source was usually taken into more consideration than the strength of neural source. In order to quantitatively evaluate the EEG source localization accuracy resulting from different BSCRs, the localization error was determined as the 3D distance between the simulated source location and the estimated source location. For each of the cortical dipole sources, the localization error was calculated by using the correct BSCR and the incorrect BSCRs, respectively. In the end, the above analysis procedure was repeated until each ratio of four BSCRs was tested as the correct ratio.

**Figure 1:**
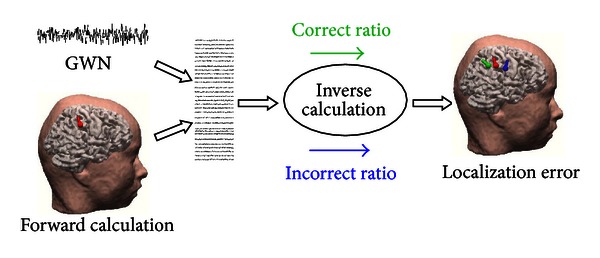
Analysis protocol for evaluating the effect of different BSCRs on EEG source localization accuracy. Gaussian white noise was added to the simulated scalp potentials to generate the noise-contaminated measurements. Localization error was calculated by comparing the distance between the simulated source and the estimated source in a realistic head volume conductor.

In order to further investigate the influence of different BSCRs on EEG source localization accuracy under the real EEG signals, the noise-contaminated EEG signals were simulated in this study. The analysis protocol of considering noise-contaminated EEG measurements, which is similar to the aforementioned analysis method of noise-free EEG measurements, is illustrated in Figure 1. Firstly, for each of the cortical dipole sources, the scalp potentials with 31-channel montage were generated by BEM-based forward calculation. Ten individual trails of Gaussian white noise (GWN) with 10 dB signal-to-noise ratio (SNR) were added to the generated scalp potentials to simulate the noise-contaminated measurements. Secondly, for each of the simulated EEG distribution, the single ECD fitting was performed to solve the inverse problem by assigning the correct BSCR and the incorrect BSCRs separately. Finally, the localization error was obtained by averaging over the 10 trails. This was able to effectively reduce the uncertainty and bias of localization error which resulted from adding Gaussian white noise to the scalp potentials.

## 3. Results

### 3.1. Noise-Free EEG Measurements

**Figure 2:**
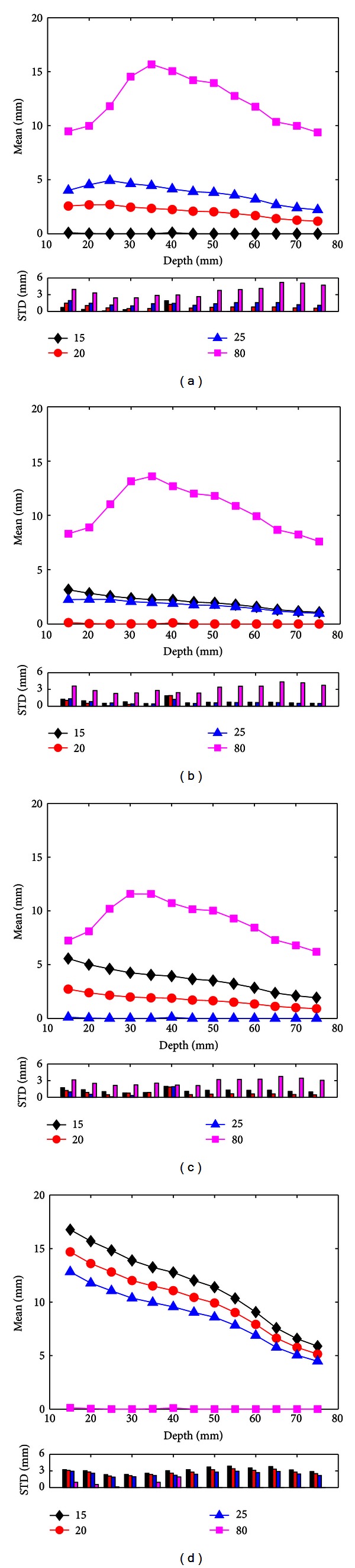
Mean and STD values of source localization errors varying with the depth of dipole sources in the case of considering noise-free EEG measurements. The BSCRs in the forward calculation were set at 15 (a), 20 (b), 25 (c), and 80 (d). The localization errors were averaged over all dipoles within each interval of 5 mm. The black, red, blue, and pink curves and vertical bars are related to the source localization results of different BSCRs used in the inverse calculation: 15, 20, 25, and 80.

The mean and standard deviation (STD) of source localization errors varying with the depth of dipole sources in the case of considering noise-free EEG measurements are illustrated in Figure 2. The depth of dipole sources was defined as the shortest distance between a single dipole source on the folded cortical surface and the skin layer in the BEM head model. The larger the value of depth is, the deeper the single dipole source is inside the brain. After the depth of cortical dipole sources was calculated, the whole range of depth was segmented into 13 intervals and each interval corresponds to 5 mm. The results shown were mean and STD values of source localization errors averaged over all dipoles within each interval. The different color curves and vertical bars depict the source localization results of four different BSCRs used in the inverse calculation. As shown in Figure 2(a), the BSCR used in the forward calculation was set at 15. When the inverse calculation was performed, the correct ratio was 15 and the incorrect ratios were 20, 25, and 80. The mean values of localization errors were approximately equal to zero when the BSCR specified in the inverse calculation was 15. This indicates that the cortical dipole sources can be accurately localized by using the correct BSCR. The mean of localization errors ranged between 1.16 and 4.92 mm with assigning the BSCR as 20 and 25 in the inverse calculation. When 80 was used as the BSCR in the inverse calculation, the mean of localization errors, which ranged between 9.39 and 15.68 mm, increased to reach a maximum and then decreased while the dipole sources became deep. The values of STD were below 1.96 mm with assigning the BSCR as 15, 20, and 25 in the inverse calculation and between 2.44 mm and 5.2 mm with assigning the BSCR as 80 in the inverse calculation. When the BSCRs were set at 20 (Figure 2(b)) and 25 (Figure 2(c)) in the forward solution, the source localization results were similar to those in Figure 2(a). In Figure 2(d), the ratio used in the forward calculation was 80. The cortical dipole sources could be accurately localized, and the values of STD were below 1.92 mm when the correct BSCR was specified in the inverse calculation. When the BSCRs used in the inverse calculation were 15, 20, and 25, the source localization errors showed the same trends that the localization errors decreased as the depth of dipole sources increased. The mean values of localization errors ranged between 4.48 and 16.77 mm, and the values of STD ranged between 1.88 and 3.87 mm.

### 3.2. Noise-Contaminated EEG Measurements

**Figure 3:**
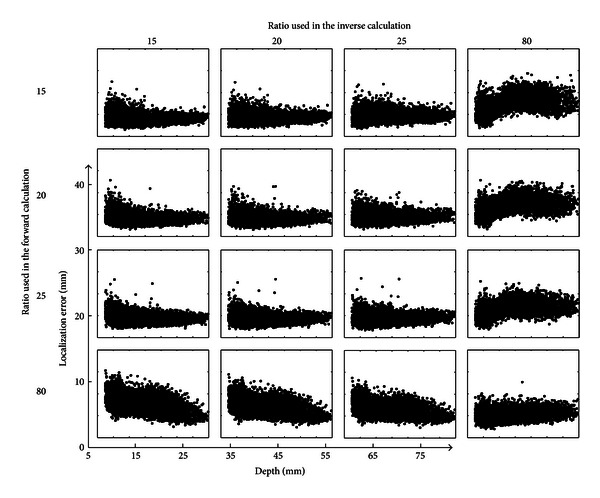
Scatterplot of the dipole localization errors obtained from four different BSCRs when considering noise-contaminated EEG measurements. Rows correspond to different BSCRs used in the forward calculation, and columns correspond to different BSCRs used in the inverse calculation.

Considering noise-contaminated EEG measurements, the scatterplot of localization errors of all cortical dipole sources with different setting of BSCRs used in the forward and inverse calculation is shown in Figure 3. Each row of the figure corresponds to a specific conductivity ration used in the forward calculation. Each column corresponds to a value of BSCR used in the inverse calculation. The dots in each subplot represent the mean values of localization errors averaged over ten scalp EEG measurements added by 10 dB noise. As shown in Figure 3, the number of shallow sources is greater than that of deep sources. For the fourth column, it can be observed that the localization errors of deep sources are higher than those of shallow sources. The localization errors of deep sources are lower than those of shallow sources for the fourth row. When the BSCRs used in the forward and inverse calculation were 15, 20, and 25, the source localization results were similar because the used BSCRs were very close to each other. The range of source localization errors was between 3.08 and 26.96 mm.

**Figure 4:**
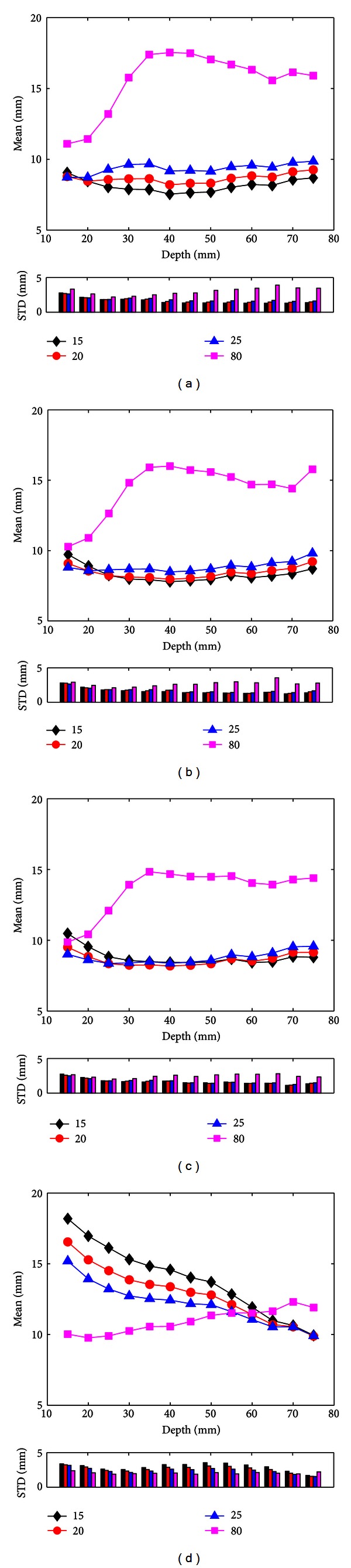
Mean and STD values of source localization errors varying with the depth of dipole sources in the case of considering noise-contaminated EEG measurements with 10 dB GWN. The BSCRs in the forward calculation were set at 15 (a), 20 (b), 25 (c), and 80 (d). The localization errors were averaged over all dipoles within each interval of 5 mm. The black, red, blue, and pink curves and vertical bars are related to the source localization results of different BSCRs used in the inverse calculation: 15, 20, 25, and 80.

The comparisons of the source localization results with noise-contaminated EEG measurements by using four different BSCRs are summarized in Figure 4. The four subplots correspond to the different BSCRs in the forward calculation: 15, 20, 25, and 80, respectively. As can be observed from Figure 4(a), the source localization results are similar to each other when the BSCRs used in the inverse calculation are 15, 20, and 25. The mean of localization errors ranged from 7.55 to 9.86 mm. However, the means of localization errors with assigning the BSCR as 80 in the inverse solution were larger than those from other BSCRs and ranged between 11.09 and 17.54 mm. The resembling trends also appeared in Figures 4(b) and 4(c). In Figure 4(d), the correct BSCR was 80 for source localization, and the mean of localization errors ranged from 9.76 to 12.31 mm when the BSCR was assigned as the correct ratio in the inverse calculation. Around the depth of 60 mm, the corresponding mean curve intersected those resulting from the incorrect BSCRs, which ranged between 9.88 and 18.19 mm. For shallow sources, the localization errors corresponding to the correct BSCR were significantly lower than those using the incorrect BSCRs. By contrast, for deep sources, the localization errors resulting from the correct BSCR were slightly higher than those from the incorrect BSCRs. From Figure 4, it can be observed that the values of STD are below 3.9 mm.

## 4. Discussion

The aim of the present study was to examine the influence of different BSCRs on EEG source localization accuracy by using an experimental protocol. In this study, we evaluated four previously used BSCRs: 15, 20, 25, and 80. Specifically, we employed both noise-free EEG measurements and noise-contaminated EEG measurements for this experiment. When four different BSCRs were assigned, the scalp EEG potentials were generated by BEM-based forward calculation for each simulated dipole source. Then, for each scalp EEG measurement, the single-ECD fitting was conducted to identify the estimated dipole sources by the correct BSCR and the incorrect BSCRs. The localization error was used to evaluate the EEG source localization accuracy between the estimated sources and the simulated sources. When using 2D head model, it had been demonstrated that there were substantial localization errors caused by the misspecification of BSCR in noise-free condition [12]. Based on a well-accepted 3D realistically shaped head model, our study had revealed that the localization errors were approximately equal to zero when assigning the correct BSCR in the inverse calculation in the case of considering noise-free EEG measurements. When the incorrect BSCRs were used in the inverse calculation, the localization errors ranged from 2 to 16 mm (Figure 2). These results indicate that the localization accuracy is also sensitive to the BSCR in EEG source reconstruction when using 3D head model.

Considering noise-contaminated EEG measurements, the localization errors were obviously larger than those using the noise-free EEG measurements as shown in Figure 4 due to the additional localization errors caused by noise. The localization errors resulting from the misspecified BSCRs were between 8 to 18 mm. Even if the correct BSCR was used in the inverse calculation, the localization errors caused by the noise were around 9 mm. In the EEG inverse problem, the localization errors are mainly caused by two parts: the modeling error (i.e., the misspecification of BSCR) and the noise. In noise-free condition, the localization errors only result from the modeling error. As a result, using the BSCR as close to actual value as possible in the inverse problem will obtain the most accurate localization results (Figure 2). However, in the realistic conditions, the EEG signals acquired in the experiment usually include the noise with certain SNR, and thus the modeling error needs to be combined with noise for considering the localization errors. As can be observed in Figure 4, for the shallow sources, the BSCR close to actual value can get more accurate source localization results than other ratios. This indicates that the localization errors of shallow sources are mainly influenced by the modeling error. On the other hand, as shown in Figures 2(a), 2(b), and 2(c), when the BSCR used in the inverse calculation was set at 80, for sources deeper than about 35 mm, the deeper the dipole location is, the smaller the localization error resulting from modeling error is. Thus, for the deep sources, the localization errors may be mainly determined by the noise for the single dipole localization. When ignoring the modeling error of the deep sources, using low BSCR will bring the small localization error for the same noise level case if the BSCR used in the forward calculation was also small. If the BSCR used in the forward problem was set at 80, using low BSCR for the inverse calculation would give small localization error for sources deeper than about 60 mm (Figure 4(d)).

**Figure 5:**
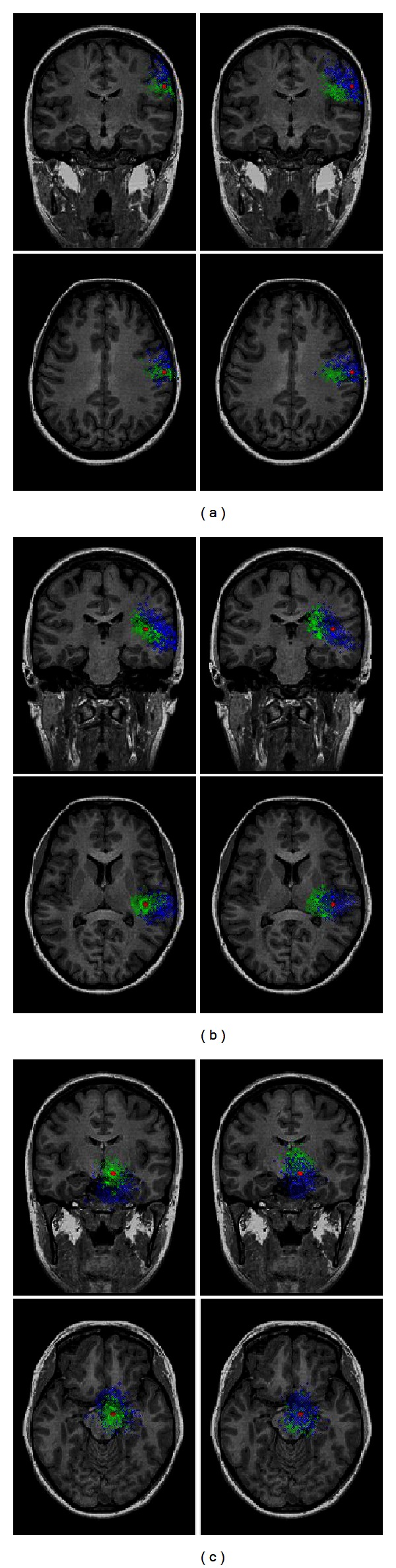
Three simulated dipole sources and the estimated sources corresponding to scalp EEG measurements contaminated by 1000 times randomly generated GWN for 31-electrode montage when the BSCRs were set at 20 (left column) and 80 (right column) in the forward calculation: (a) shallow source; (b) middle source; (c) deep source. The red point represents the simulated dipole source. The green points denote that the BSCR used in the inverse calculation was 20. The blue points denote that the BSCR used in the inverse calculation was 80.

In order to further elaborate the influence of the modeling error and the noise on EEG source localization error, the localization results of shallow, middle, and deep dipole source for 31-electode montage in the situation of GWN (1000 runs of noise generation) are illustrated Figure 5. Here, the depth ranges of shallow source, middle source, and deep source were defined as from 5 to 25 mm, from 25 to 50 mm, and from 50 to 75 mm. The mean and STD values of the localization errors for three different dipole sources for different BSCRs with 31-electrode montage are shown in Table 1. For shallow source (Figure 5(a)), when the BSCR used in the forward calculation was set at 20 (the left column of Figure 5(a)), the localization accuracy resulting when the BSCR used in the inverse calculation was set at 20 (green points) was better than those resulting when the BSCR was set at 80 (blue points). When the BSCR used in the forward calculation was set at 80 (the right column of Figure 5(a)), the correct BSCR still resulted in better source localization accuracy. The localization results of middle source (Figure 5(b)) were comparable to those of shallow source. Furthermore, the difference of localization results between the correct BSCR and the incorrect BSCR almost diminished when assigning the BSCR as 80 in the forward computation. For deep source shown in Figure 5(c), the localization accuracy related to low BSCR was better than that of high BSCRs in spite of the BSCRs used in the forward calculation. This is mainly because the low BSCR will lead to the small localization error for the same noise level case when solving the EEG dipole source localization problem. These results are consistent with the observations of Figure 4. On the other hand, the estimated dipole sources are localized above the simulated dipole sources when the used BSCR is larger than the correct BSCR, as indicated in Figure 5(a). The estimated dipole sources are localized below the simulated dipole sources when the used BSCR is smaller than the correct BSCR for shallow sources. For deep sources, the opposite situation can be observed in Figure 5(c) since the blue area in the left column is below the red dot and the green area in the right column is above the red dot. For middle sources in Figure 5(b), the estimated dipole sources are lateral to the simulated dipole sources when using the BSCR larger than the correct value and the estimated dipole sources are mesial to the simulated dipole sources when using the BSCR smaller than the correct value.

**Figure 6:**
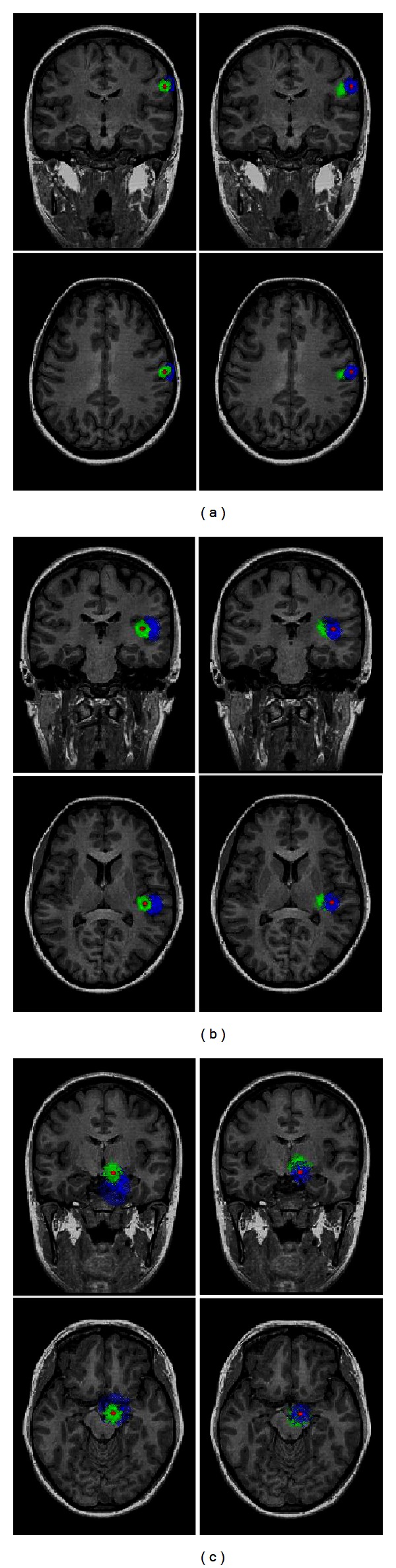
Three simulated dipole sources and the estimated sources corresponding to scalp EEG measurements contaminated by 1000 times randomly generated GWN for 128-electrode montage when the BSCRs were set at 20 (left column) and 80 (right column) in the forward calculation: (a) shallow source; (b) middle source; (c) deep source. The red point represents the simulated dipole source. The green points denote that the BSCR used in the inverse calculation was 20. The blue points denote that the BSCR used in the inverse calculation was 80.

In addition to BSCR and depth of dipole source, recent studies had suggested that number of scalp electrodes also had significant impact on EEG source localization accuracy [21, 22]. In this study, we further investigated the effect of the 31-electrode montage and the 128-electrode montage on EEG source localization. The 128-electrode montage was also configured on the modified 10/20 system [15]. The localization results of shallow, middle, and deep dipole source for 128-electrode montage in the situation of GWN are illustrated Figure 6. The mean and STD values of the localization errors for different BSCRs with 128-electrode montage are shown in Table 2. In contrast to the source localization performance of 31-electrode montage, it can be observed that the results of 128-electrode montage have obviously less localization errors than those of 31-electrode montage for three dipole sources. With an increasing number of electrodes, scalp EEG signals can provide an enhanced spatial resolution and more information of electrical activity distribution inside human brain [23, 24]. Our results testified the possibility of accurately localizing brain electrical activity sources when using a high density electrode montage to solve the EEG dipole source localization problem. In terms of the relationships between different BSCRs and EEG source localization accuracy, the cortical dipole sources can be accurately localized by the correct BSCR for all three dipole sources with different depths. This indicates that the localization errors resulting from the noise can decrease along with the increase of EEG electrode number for deep dipole sources. Therefore, when using high density electrode montage, the localization errors are not primarily generated by the noise but determined by the modeling error for the deep sources. As mentioned before, obviously, the localization errors are also mainly determined by the modeling error for the shallow sources.

## 5. Conclusions

In summary, we have investigated the influence of different BSCRs on EEG source localization accuracy in a realistically shaped head volume conductor by using both noise-free EEG measurements and noise-contaminated EEG measurements. The relationships between different BSCRs and EEG source localization can be mainly affected by the noise, the number of scalp electrodes, and the depth of the brain electrical activity source. In the present study, it can be observed that substantial source localization errors may occur if the BSCR is misspecified for EEG inverse source imaging. Our results demonstrate the significance of accurate specification of BSCR for EEG dipole source localization problem. In the case of considering noise-free EEG measurements, the cortical dipole sources could be accurately localized when the conductivity ratio used in the inverse calculation was set at the correct BSCR. The specification of incorrect BSCRs would result in substantial localization errors which ranged between 2 and 16 mm. In the case of considering noise-contaminated EEG measurements, the source localization errors ranged between 8 and 18 mm in spite of the BSCRs used in the inverse calculation. Therefore, EEG source localization can be accurately achieved only if the correct BSCR and the EEG scalp signals with high SNR are used in the inverse calculation. In addition, because EEG signals can provide more information of brain electrical activity when increasing the number of electrodes, the localization results of 128-electrode montage have obviously less errors than those of 31-electrode montage for different dipole sources.

One limitation of this study was that the actual source was assumed as a single equivalent current dipole located over the cortex and perpendicular to the folded cortical surface reconstructed from a human subject's magnetic resonance (MR) image of the head. In fact, most generators of EEG activity are distributed sources, which should be modeled as a dipole sheet extending over many square centimeters of cortical surface. Therefore, this research will further investigate the effect of BSCR on EEG source localization accuracy by using an extended dipole sheet in the future.

## Figures and Tables

**Table 1:** Mean and STD values of the localization errors (mm) for shallow, middle, and deep dipole source for different BSCRs with 31-electrode montage when the noise-contaminated EEG measurements are simulated by 1000 times randomly generated GWN.

Inverse	Forward of shallow source		Forward of middle source		Forward of deep source	
	20	80	20	80	20	80
20	9.6 ± 4.6	16.8 ± 5.7	9.0 ± 4.4	13.2 ± 5.1	9.6 ± 4.3	12.1 ± 5.0
80	12.2 ± 5.7	10.7 ± 5.5	17.3 ± 7.0	11.9 ± 5.7	16.6 ± 6.7	13.3 ± 5.7

**Table 2:** Mean and STD values of the localization errors (mm) for shallow, middle, and deep dipole source for different BSCRs with 128-electrode montage when the noise-contaminated EEG measurements are simulated by 1000 times randomly generated GWN.

Inverse	Forward of shallow source		Forward of middle source		Forward of deep source	
	20	80	20	80	20	80
20	2.0 ± 1.0	9.9 ± 1.7	3.2 ± 1.4	9.0 ± 2.1	4.5 ± 2.0	8.1 ± 2.8
80	6.7 ± 1.3	3.0 ± 1.3	9.7 ± 1.8	4.5 ± 2.1	11.8 ± 4.1	6.4 ± 2.7
